# Tapping to hip-hop: Effects of cognitive load, arousal, and musical meter on time experiences

**DOI:** 10.3758/s13414-020-02227-4

**Published:** 2021-01-29

**Authors:** Clemens Wöllner, David Hammerschmidt

**Affiliations:** University of Hamburg, Institute of Systematic Musicology, Neue Rabenstr. 13, 20354 Hamburg, Germany

**Keywords:** Attention, Prospective duration estimation, Passage of time, Dual task, Tapping, Metrical levels

## Abstract

Experiences of time vary intra- and interindividually, depending on factors such as attentional resource allocation and arousal. Music as a temporal art that is structured by multiple temporal layers is ideal for investigating human time experiences. The current study used examples of hip-hop music that varied in arousal but were constant in tempo. Participants judged the passage of time to be quicker when cognitive load was high in a dual-task condition, and perceived duration to be shorter when performing a concurrent motor task (tapping along with the music). Perceived musical arousal did not affect subjective time. Attending to a higher metrical level by tapping with half notes resulted in shorter duration estimates and a quicker passage of time, compared to tapping with eighth notes of the same music. Results were not influenced by spontaneous motor tempo, musical expertise, preference or familiarity with the music. Taken together, these findings indicate consistent effects of cognitive load and attention to meter on time experiences.

## Introduction

There has been a remarkable slowing-down in musical tempo in recent years. The beat-per-minute (bpm) rates for the most popular music on Spotify was slower by 23 bpm, resulting in a new average tempo of 90.5 bpm in the years 2012–2017. It has been speculated that this may reflect a change in mood and attitude to time, or represents the dominance of hip-hop over other musical genres (Leight, 2017). Indeed, the widespread popularity of hip-hop comes with slower tempi. Previous research suggests that tempo – that is the number of perceived events in a given time span – influences arousal and subsequently experience of time (Droit-Volet, Ramos, Bueno, & Bigand, 2013). It is less known whether listening to different music at the same tempi affects time experiences. The aim of the current study was to investigate how time is perceived in relatively slow hip-hop music by experimentally varying musical arousal and cognitive load. In particular, listeners were asked to only listen to music, to focus their attention on two different metrical levels in a sensorimotor synchronization task, or to carry out a concurrent working memory task that increased cognitive load. A further aim was to relate listeners’ time experiences to their spontaneous motor tempo, their preference as well as familiarity with the musical genre.

When investigating how individuals perceive time in filled-duration intervals such as music, it is possible to focus on (a) factors in the stimulus material, for instance musical tempo, intensity, perceived arousal and complexity, or (b) factors in the listeners including their experiences and emotional states, or (c) the listening context including concurrent activities. Regarding the first perspective, research has shown that subjective duration estimates can be influenced by elements such as the melodic structure in music that lead to temporal expectations about the occurrence of phrase endings (Boltz, 1989; Drake, Jones, & Baruch, 2000). Complexity in the composition should increase the number of events perceived, resulting in time to be judged longer (Bueno, Firmino, & Engelman, 2002). In this research, participants listened to symphony movements from Mahler or Berio. In prospective time reproductions, in which individuals are aware beforehand that they should reproduce a time interval (cf. Block & Zakay, 1997), they underestimated the duration of the Mahler piece, while overestimating Berio’s music, which was higher in complexity. Increases in musical tempo also led to more events perceived. In a study on waiting time perception, Oakes (2003) found that participants underestimated the duration when listening to background music at slow tempi, and also seemed to prefer slow music, while fast-tempo music resulted in overestimations of waiting time. In other words, when individuals processed more events in fast music, they believed that time lasted longer. These results were confirmed in further experiments (Droit-Volet et al., 2013; Hammerschmidt & Wöllner, 2020), suggesting that fast musical stimuli are associated with increased perceived arousal, and time seems to last longer than for slow music. Nevertheless, when comparing piano to orchestra versions of the music at the same tempi, perceived arousal was judged to be higher in the latter, but this did not affect time judgements (Droit-Volet et al., 2013). In audiovisual film scenes, music compared to no music increased the physiological arousal of participants, resulting in longer duration estimates (Wöllner, Hammerschmidt, & Albrecht, 2018). It is not yet known whether perceptions of durations increase because more events are processed in fast stimuli, or because such stimuli are also more arousing. Apart from physiological arousal of the participants, the perceived arousal of the stimuli should thus be controlled in relation to tempo. Changes in physiological and perceived arousal may lead to different results.

Regarding the perspective of the individual, few studies varied listeners’ arousal and emotions in order to analyse temporal experiences. Jakubowski, Halpern, Grierson, and Stuart (2015) manipulated individual arousal and subsequently asked two groups of participants to choose an appropriate tempo of well-known songs. While one group had stayed still and solved a cognitive task, the other group had engaged in a physical exercise (jogging in place) resulting in a higher heart rate. The high-arousal group subsequently selected faster tempi for the songs, which suggests that their tempo selection matched the higher number of pulses that were emitted in this group’s putative inner clock pacemaker, and that subsequently influenced their preferred event rates. The physical exercise may also have increased participants’ spontaneous motor tempo, which has been shown to correlate with preferred tempi in selection tasks (see McAuley, Jones, Holub, Johnston, & Miller, 2006). Using an experience-sampling method, in which participants gave time and arousal judgements in their daily life repeatedly, Droit-Volet, Trahanias, and Maniadakis (2017) found that the level of individuals’ subjective arousal was only related to perceived passage of time, but not to verbal duration estimates or reproductions. Hence, factors in the participants may lead to different findings as compared to analysing factors in the stimulus material, as stated above. A further duration-estimation study, on the other hand, used visual stimuli and different physical exercises, and found that participants’ higher subjectively felt arousal resulted in longer duration estimates, while there was no effect of increased heart rate on time judgements (Schwarz, Winkler, & Sedlmeier, 2013).

Arousal has been suggested to influence the pacemaker in an internal clock (Burle & Casini, 2001; Gibbon, 1991; Noulhiane, Mella, Samson, Ragot, & Pouthas, 2007; Wearden, 2008). The internal or central clock is thought to include a pacemaker that emits pulses at different rates, which are then counted by an accumulator during time judgements. If more pulses are emitted, such as in states of increased arousal, a higher number of pulses is accumulated in a given time span. In comparison to a reference duration stored in long-term memory, the time span with more pulses should then be experienced to last longer. Neural-imaging research suggests that the insula and areas in the temporal and parietal lobes are involved in these accumulation processes (Wittmann, Simmons, Aron, & Paulus, 2010). While definite evidence for a single pacemaker is still lacking, and it can be assumed that a network of different brain areas is involved (van Wassenhove, 2016), the psychological pacemaker-counter model has high predictive power particularly for durations exceeding 3 s (i.e., the psychological present).

There are instances when not all pulses are counted by the accumulator. Prospective estimates of time intervals require attentional resources to the duration of a stimulus or empty interval, which in humans is supposedly facilitated by an attentional gate in the internal clock model that regulates the passage of pulses (Block, Hancock, & Zakay, 2010). If attention is diverted, for instance by carrying out a second task, fewer pulses are counted in the accumulator, hence time appears to be shorter. In this regard, the accumulator can be seen as a function of working memory with limited capacities (see Polti, Martin, & van Wassenhove, 2018). Since the same executive resources are required for concurrent tasks, the higher cognitive load leads to shorter duration experiences in these dual-task interferences. In other words, carrying out a secondary task may distract attention away from perceiving time, and hence individuals are more prone to underestimate durations. This leads to the somewhat paradoxical situation that high cognitive load decreases perceived durations, while high arousal increases perceived durations. Research thus needs to vary cognitive load in a way that does not increase arousal, and vice versa. The current study aimed at dissociating the effects of cognitive load and perceived arousal on time judgements with a musical task.

Manipulating cognitive load typically involves a secondary task that increases demands on working memory and attention. For musical time judgements, this addresses the third perspective from above: the listening context or listening condition. In a previous music dual-task experiment, listeners underestimated the duration of time intervals and were less accurate when they were asked to detect targets in a melody, compared to listening only (Brown & Boltz, 2002). Attending to the secondary task, that is detecting the targets, required a split of attention to both the duration estimation and the target detection. Furthermore, the higher the number of events in a task, indicating higher working memory load, the less individuals are able to reproduce short interval durations (Teki & Griffiths, 2014). Polti et al. (2018) compared single-task with dual-task duration estimations while asking individuals to judge the duration of empty intervals lasting 30, 60 or 90 s, as shown by visual signals on a screen. The dual task consisted of a visual working memory task and was meant to interfere with duration estimation. As a result, individuals judged interval durations to be shorter when attention was split in the dual-task condition, and more so for longer time intervals and correspondingly higher working memory load. These findings corroborate the attentional-gate model for prospective timing with regard to cognitive load (Block et al., 2010); durations thus appear to be shorter with increased demands on attention.

The current study varied both attentional resources allocated to a specific task (cognitive load) and perceived arousal. A prospective timing task was chosen in which participants estimated stimulus durations and judged the perceived passage of time, indicating how quickly time had passed. Previous research has shown that both judgements are related to each other for longer durations (2–8 min), but not for shorter ones (3–33 s; Droit-Volet et al., 2017), and another study found no relationships between duration estimates and passage-of-time judgements (Droit-Volet & Wearden, 2016). These judgements thus refer to different evaluation processes in timing tasks.

Apart from arousal and attention, there are further factors that may influence perceived time in filled intervals such as music. One of these factors is familiarity, so that listeners estimate wait lengths to be shorter with familiar background music compared to an unfamiliar one (Bailey & Areni, 2006). Presumably, participants directed more attention to the familiar music in this study, and thus diverted less attention to the duration-estimation task. A meta-analysis, on the other hand, does not support familiarity effects for prospective (non-musical) timing tasks (Block et al., 2010). In addition, preference for musical genres may influence duration estimates, suggesting that durations of preferred music seem to be shorter (Lopez & Malhotra, 1991). It can be speculated that listeners were more absorbed in the music they liked, and thus focused less attention on time judgements.

Recent research also provides evidence that duration estimations depend on the the metrical level in music individuals synchronize to (Hammerschmidt & Wöllner, 2020). Listeners tapped to the half-note, quarter-note and eighth-note metrical level of the same four-voice percussion patterns, or listened only to the patterns in a control condition. Stimulus duration was judged shortest in the half-note tapping condition, indicating that time intervals appeared to be less filled when attending to a higher metrical level. When listeners can choose the metrical level they wish to synchronize with, some studies suggest that musical training (Drake, Penel, & Bigand, 2000) or cultural familiarity with music (Drake & El Heni, 2003) led listeners to synchronize with higher levels, hence to perceive larger structures. Other studies did not find effects of musical expertise for a specific musical genre (Snyder & Krumhansl, 2001), or a variety of musical genres for which participants tapped at different rates, independently from musical expertise (McKinney & Moelants, 2006). Perceiving musical meter as a hierarchically organized and recurring sound pattern is based on expectations of events in time (London, 2002) as well as sound characteristics of the music (e.g., Burger, London, Thompson, & Toiviainen, 2018), and may shape perceptions of musical durations (Hammerschmidt & Wöllner, 2020). Based on musicians’ higher accuracy and consistency in time reproduction (Grondin & Killeen, 2009) and temporal synchronization tasks (Repp, 2010), there is reason to assume that the experience of musical time is also influenced by expertise.

In the current study, we investigated the experience of time in relation to attentional demands (i.e. cognitive load), perceived arousal, and musical expertise. Participants listened to hip-hop music that varied in perceived arousal as confirmed in a pilot study and with a further manipulation check. They synchronized with the music at a higher metrical level (half notes, longer inter-onset intervals) or a lower one (eighth notes, shorter inter-onset intervals). While the tempo of the music (measured in bpm) was kept constant, attentional resource allocation was varied by three conditions (in ascending order): listening only, tapping along with one of the two metrical levels, or solving a concurrent mathematical task. It was assumed that attention was diverted most in the dual-task condition with highest cognitive load (Block et al., 2010), while the tapping task should distract participants less from the music (medium cognitive load), since tapping to the beat is a common activity in music listening. In the listening-only condition, participants should be the least distracted and thus allocate more attention to time (low cognitive load). In addition, we tested whether musical expertise, familiarity, and preference for the music had an impact on time perception. Finally, participants’ preferred motor tempo, that is their spontaneous tapping rate without any external stimulus (cf. McAuley et al., 2006), may influence how they experienced the beat of the music and musical time. In summary, we assumed that:

1. Duration would be underestimated, and passage of time would be quicker in the dual-task condition with the highest cognitive load (Block et al., 2010), and relatively longer in the medium and low cognitive-load conditions;
2. High arousal music would lead to longer duration estimates and slower passage of time (Droit-Volet et al., 2013);
3. Attending to a higher metrical level would shorten perceived time intervals and lead to quicker passage of time (Hammerschmidt & Wöllner, 2020);
4. Preferred motor tempo, musical expertise, preference and familiarity may influence experienced time (Bailey & Areni, 2006; Lopez & Malhotra, 1991). Since results in previous literature are inconsistent, we do not postulate a direction of the potential influences.

## Methods

### Participants

A total of 30 individuals took part in the study. An a-priori power analysis using G*power (Faul, Erdfelder, Lang, & Buchner, 2007) had resulted in 28 participants needed for *α* = .05, 1-*β* = .95, and an estimated effect size of *f* = .25. One participant with no musical training had difficulty in synchronizing with the hip-hop stimuli and was subsequently excluded from analysis. The remaining 29 participants (18 female, 11 male) had a mean age of 25.17 years (*SD* = 6.44). Participants were mostly recruited from the Institute of Systematic Musicology and had received instrumental or voice lessons with a teacher for *M* = 10.14 years (*SD* = 6.53; *Mdn* = 10.00). They took part in accordance with the guidelines of the local Ethics Committee and were remunerated for their participation.

### Musical examples

Four hip-hop songs were selected for the main experiment, based on the results of a pilot study. Two of the songs were low in perceived arousal, and two were high in perceived arousal (Table 1). The pilot study had included a total of 18 excerpts from hip-hop songs without lyrics, with an original mean tempo of 93.16 bpm (*SD* = 1.95). The musical examples were slightly adjusted in tempo using Ableton Live 10, so that all of them were finally at 93 bpm (equivalent to an inter-onset interval of 645.16 ms). In the pilot study, 20 participants (age: *M* = 21.15 years, *SD* = 3.03; five female, 14 male, one other; years of musical training: *M* = 7.98, *SD* = 4.84) had judged the perceived arousal on a seven-point scale (“How aroused/excited or calm was the musical example?”; 1 = very calm, 7 = very aroused/excited). Based on these judgements, two musical examples with the lowest arousal ratings and relatively small standard deviations and two with the highest ratings were selected. They differed significantly from each other in perceived arousal, *F*(3, 57) = 59.55, *p* < .001, *η*_*P*_^*2*^ = .76; in posthoc tests (Bonferroni corrected), low- and high-arousal examples differed at *p* < .001, the two low-arousal examples did not differ from each other (*p* = .636), and the two high-arousal examples differed slightly (*p* = .044). Note events per second, spectral flux (indicating the change of timbre over time), spectral centroid (related to the perceived sound brightness), and RMS energy (as a measure of intensity, using windows of 25 ms with 50% overlap) were analysed using the MIRtoolbox for Matlab (Lartillot, Toiviainen, & Eerola, 2008). Three duration versions of each example were produced, resulting in eight, ten, or 12 bars (4/4 meter each) without lyrics.

**Table 1:** Selected musical examples

Arousal	Producer, artist, song	Album, release year	Original bpm* (quarter notes)	Notes/Sec	Spectral flux (100–200 Hz)	Spectral centroid (Hz)	RMS energy *M (SD)*
Low	RZA, Wu-Tang Clan: C.R.E.A.M.	Enter the Wu-Tang, 1993	90	4.62	4.72	1,843.54	0.12 (0.08)
Low	Havoc, Mobb Deep: Hell on Earth	Hell on Earth, 1996	92	5.62	3.60	2,797.54	0.22 (0.16)
High	Kanye West, Talib Kweli: Get By	Quality, 2002	90	6.07	5.27	1,943.14	0.25 (0.13)
High	Damian Marley, Nas & D. Marley: Nah Mean	Distant Relatives, 2010	94	6.62	4.21	3,215.73	0.26 (0.15)

*In the experiment, tempo was adjusted for all examples to 93 bpm

### Design and procedure

**Fig. 1:**
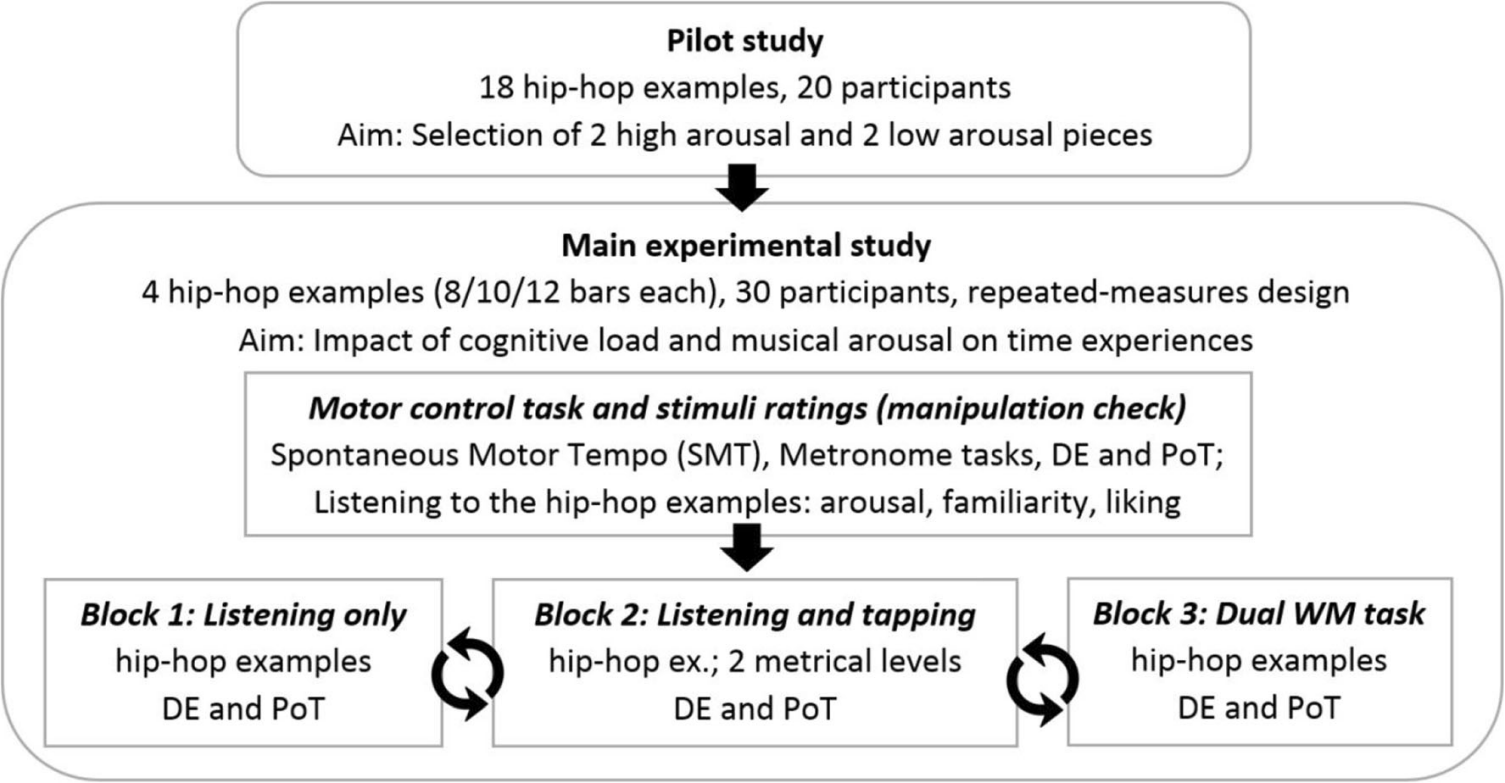
Overview of the pilot and main study. In each main experimental task, participants provided duration estimations (DEs), and indicated the perceived passage of time (PoT)

In a repeated-measures design, two arousal conditions of stimuli (low, high) were presented to participants, who completed three experimental task conditions with increasing cognitive load (listening only, listening and tapping, dual task). In the tapping condition, participants were asked to focus on two metrical levels (eighth notes, half notes). Figure 1 provides a schematic overview of the whole study.

The experiment was conducted using the OpenSesame platform (Mathôt, Schreij, & Theuwes, 2012), and stimuli were played back via Ableton Live using a Focusrite Scarlett 2i2 audio interface and Beyer Dynamics DT-880 Pro headphones. After answering demographic questions, participants tapped freely on a MIDI bop-pad (McMillen Instruments) at a tempo that felt most natural to them, in order to record their spontaneous motor tempo (SMT). They subsequently listened to a metronome at 46.5, 93 or 186 bpm in a control condition without music for 23 s, and were asked to either tap along with the metronome or to listen only. After each trial, participants estimated how long the trial had lasted (in seconds) and judged the subjectively perceived passage of time (PoT, see Droit-Volet et al., 2017). The reason for including the control task without music was to see whether the mere motor activity of tapping, compared to listening only to the metronome, affected time judgements. Participants then listened to the actual hip-hop stimuli and indicated on several seven-point scales: (a) the level of arousal perceived, (b) their familiarity with the musical example, and (c) their liking of it (preference). The arousal ratings served as a manipulation check to see whether perceived arousal matched the selection based on the pilot study.

The main experimental tasks consisted of three experimental blocks that were presented in counterbalanced order. In Block 1, participants listened only to the hip-hop examples at durations of eight, ten, or twelve bars in (pseudo-)randomized order (total: 12 trials), with the rule that no successive trials consisted of the same hip-hop example. The respective durations were 20.645 s, 25.806 s and 30.968 s. Participants judged after each stimulus how long it had lasted (duration estimation), and indicated on a seven-point scale how quickly time had passed (PoT).

In Block 2, participants were presented with the same musical examples as in Block 1 or Block 3, this time preceded by a cowbell-like count-in sound at two beat rates in order to indicate the metrical level they should attend to. The count-in sounds each contained eight sounds and lasted 2.581 s for the eighth-note metrical level, and 5.162 s for the half-note metrical level. The inter-onset interval (IOI) for the eighth-note metrical level was 323 ms, and for the half-note level 1,290 ms, which is equivalent to the 186 bpm and 46.5 bpm used in the control task when tapping with a metronome. Number of IOIs ranged from 16 (half-note level for durations of eight bars) to 96 (eighth-note level for durations of 12 bars). Since the four hip-hop examples contained a steady pulse (hence equidistant IOIs) and had been adjusted in overall tempo, durations and IOIs were similar for all of them. Following practice examples with other hip-hop music at the same tempo and both metrical levels, participants listened to the stimuli while tapping along with the metrical level as specified by the count-in sounds (total: 24 trials). They estimated the duration of the musical example, and judged perceived PoT.

In Block 3, participants completed the dual-task condition that included the same stimuli as before, judgements of duration and PoT, and additionally a concurrent working memory math task in order to increase cognitive load (Block et al., 2010). While listening to the music, participants counted backwards in steps of three from three-digit numbers such as 429 or 907, and typed in their result after the end of the stimulus.

At the end, participants tapped again freely without music at a tempo of their choice in order to indicate SMT. The total duration of the experiment was 30–50 mins, depending on individual participants.

### Data analysis

First it was analysed whether participants had adequately tapped along with the intended metrical level. If an inter-tap interval was above or below 25% of the actual IOI, it was not considered to be aligned to the meter. Following this criterion, all but one participant succeeded in tapping to the eighth-note or half-note level. The one participant was excluded because 41.67% of this participant’s tapping trials were invalid, leading to a total of 29 participants included in analysis as mentioned above. The duration estimation and PoT responses were scanned for outliers beyond three SDs from the sample mean. For duration estimation, five cases (1.44%) were excluded for the listening-only condition, seven (1.01%) for the listening-tapping condition, and eight (2.30%) for the dual-task. For PoT, only one case (0.29%) was excluded for the dual-task condition. Missing values were not interpolated, since collapsing of data across trials and cases resulted in valid dependent variables for all 29 participants.

The three different durations (8, 10, 12 bars) were clearly reflected in participants’ duration estimations, collapsed over musical excerpts, *F*(1.54, 43.05) = 58.10, *p* <. 001, *η*_*P*_^*2*^ = .68 (Greenhouse-Geisser corrected), all post hoc comparisons (Bonferroni) were significant at *p* < .001. Hence participants differentiated between durations based on the different numbers of bars. Since duration estimations were correlated (all *r* > .89, *p* < .001), they were collapsed across durations for further analyses. Duration estimation ratios were calculated by dividing participants’ subjective estimations in seconds by the corresponding objective clocktime values in seconds.

Furthermore, participants reliably differentiated between the low- and high-arousal musical examples, *t*(28) = 15.04, *p* < .001, Cohen’s *d*_*z*_ = 2.97, and thus perceived the arousal in the examples comparably to the participants in the pilot study. Hence the manipulation check was successful and perceived arousal differed indeed for the stimuli. In the dual-task condition, the math task was considered to be correct when the response value was lower than the original value and a multiple of 3. Successful task completion (number of correct trials) was not related to duration estimations or PoT, for neither low- nor high-arousal music (all *r* < .13, all *p* > .315).

Repeated-measures ANOVAs were calculated, using Greenhouse-Geisser corrections if the sphericity assumption was not met, and Bonferroni corrections were used for multiple post hoc comparisons. Two multiple linear regressions were calculated to indicate whether duration estimations and PoT could be predicted by participants’ mean SMT, expertise, familiarity and preference.

## Results

### Cognitive load and arousal

**Fig. 2:**
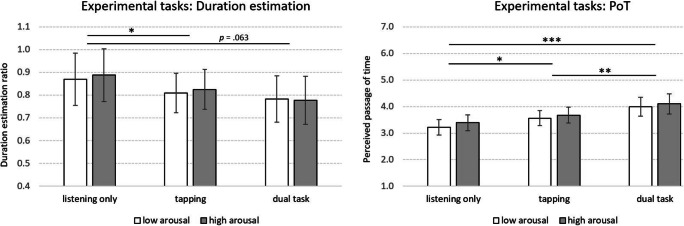
Effects of experimental tasks (listening only, tapping and listening, dual task) and musical arousal (low, high) on duration estimation ratios (1 equals clocktime) as well as perceived passage of time (PoT; 7 indicates “very fast”; error bars represent 95% confidence intervals). Asterisks indicate significant differences: * *p* < .05, ** *p* < .01, *** *p* < .001

It was expected that the higher the cognitive load, the more attention would be diverted and the shorter the perceived durations. Listening only should involve lowest cognitive load, while focusing on the two metrical levels in the tapping condition (collapsed for this analysis since metrical levels were highly correlated, all *r* > .85, *p* <.001), or carrying out mental calculations would result in a higher load. A two-way repeated-measures ANOVA on experimental task condition (listening only, listening and tapping, dual task) and arousal (low, high) resulted in a main effect for task condition, *F*(1.34, 37.55) = 5.19, *p* = .020, *η*_*P*_^*2*^ = .16, but not for arousal *F*(1, 28) = .88, *p* = .358, *η*_*P*_^*2*^ = .03, with no interaction (Fig. 2). Post hoc comparisons indicate that time was perceived to last longer while listening only compared to tapping along with the music (*p* = .043), and marginally longer for listening only compared to the dual-task condition (*p* = .063).

For perceived passage of time (PoT), the ANOVA resulted in an effect for experimental task, *F*(2, 56) = 15.05, *p* < .001, *η*_*P*_^*2*^ = .35, but not for arousal, *F*(1, 28) = 2.78, *p* = .106, *η*_*P*_^*2*^ = .09, with no significant interaction (Fig. 2, right panel). Post hoc analyses show that PoT was quicker in the dual-task condition compared to listening only (*p* < .001) and tapping (*p* = .006), and time passed more quickly while tapping rather than listening only (*p* = .036).

### Metrical levels and arousal

**Fig. 3:**
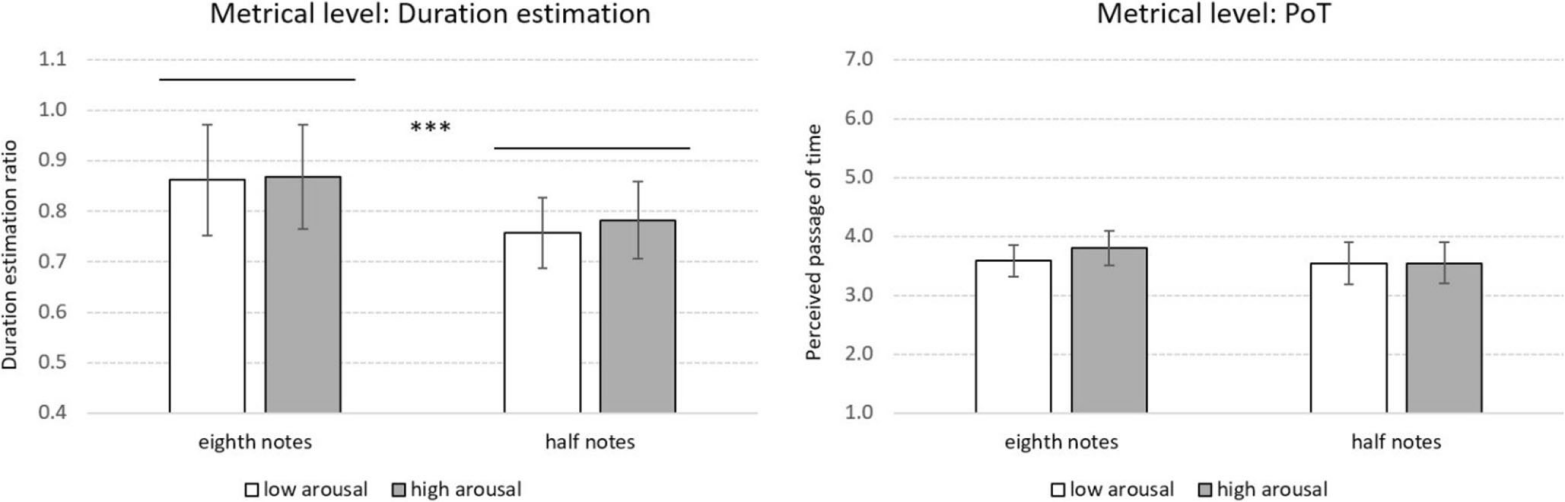
Effects of attending to different metrical levels (eighth notes, half notes) and arousal (low, high) on duration estimation ratios and perceived passage of time (PoT; error bars represent 95% confidence intervals)

Did the metrical level participants attended to influence how they perceived time? A two-way repeated-measures ANOVA on metrical tapping level (half notes, eighth notes) and stimulus arousal (low, high) resulted in a significant main effect for metrical level, *F*(1, 28) = 15.35, *p* < .001, *η*_*P*_^*2*^ = .35. As can be seen in Fig. 3 (left panel), participants underestimated stimulus duration across conditions, but significantly more so when tapping along with the half-note metrical level. There was no effect of arousal, *F*(1, 28) = 1.68, *p* = .206, *η*_*P*_^*2*^ = .06, and no interaction.

A similar two-way ANOVA was calculated for perceived PoT, which resulted in no significant effects for metrical level, *F*(1, 28) = 1.81, *p* = .189, *η*_*P*_^*2*^ = .06; arousal, *F*(1, 28) = 1.93, *p* = .175, *η*_*P*_^*2*^ = .07; and no interaction.

In the motor control condition, it was investigated whether the mere activity of tapping with a metronome at different rates had influenced duration estimations and PoT, independently from the music. A repeated-measures ANOVA on duration estimation resulted in no effect of beat rate, *F*(2, 52) = 1.21, *p* = 0.31, *η*_*P*_^*2*^ = 0.04. There was an effect for tapping versus no tapping, *F*(1, 27) = 10.35, *p* = .003, *η*_*P*_^*2*^ = 0.29, such that participants perceived trials to last shorter when they had tapped. For PoT, the ANOVA yielded an effect of beat rate, *F*(1.65, 44.50) = 4.49, *p* = .016, *η*_*P*_^*2*^ = 0.14, and post hoc tests showed that time passed more quickly for 93 bpm compared to 46.5 bpm. PoT was also higher for tapping as compared to no tapping, *F*(1, 28) = 11.20, *p* = .002, *η*_*P*_^*2*^ = 0.29. No interactions occurred.

### Spontaneous motor tempo, familiarity, preference, expertise

Regarding SMT, participants tapped freely at a mean IOI of 715 ms (*SD* = 178) at the beginning of the experiment, and at a statistically comparable rate of 684 ms (*SD* = 337) at the end, *t*(28) = 0.587, *p* = .562, *d* = .186. It should be noted that mean SMT was in the tempo range of the hip-hop examples used in the study (at 93 bpm, 645.16 ms IOI). Pre- and post-SMT were highly related, *r* = .562, *p* = .003. While participants’ overall familiarity with the musical examples as judged on a seven-point scale was low (*M* = 1.37, *SD* = 1.40), they did not dislike the music (*M* = 3.40, *SD* = 1.12), which was also judged on a seven-point scale.

A multiple linear regression analysis was carried out on overall duration estimation (collapsed across the three experimental tasks), with participants’ years of musical training, familiarity with the music, preference, and mean SMT as predictor variables, *R*^*2*^ = 0.06, *F*(4, 24) = 0.40, *p* = .804, *η*^*2*^ = .06 (all *β* < 0.11). A comparable linear regression on PoT resulted in *R*^*2*^ = 0.16, *F*(4, 24) = 1.11, *p* = .373, *η*^*2*^ = .16; with *β* = 0.41 for preference approaching significance (*p* = .058), all other *β < 0.20*. Hence these variables could not significantly predict participants’ overall duration estimation or PoT, as only preference for the music was marginally related to faster perceived passage of time.

## Discussion

Since music as a temporal art fills time intervals with varying levels of complexity and arousal, characteristics of the music may change experiences of time. While previous research on duration judgements investigated the impact of cognitive load or perceived arousal separately, no study so far has attempted to disentangle both with real musical examples. In accordance with the main hypothesis of the current study, engaging in concurrent working memory tasks resulted in faster perceived passage of time due to higher cognitive load. Correspondingly, perceived durations decreased with higher load, hence the time intervals appeared to be shorter, but only marginally so for duration estimates in the highest load condition (two-tailed). Tempo was kept constant for all musical examples and was thus dissociated from perceived arousal of the stimulus, which in turn did not significantly modulate time experiences. Attending to different metrical levels in the hip-hop music clearly affected duration estimates, which were shorter when individuals synchronized with half notes compared to eighth notes of the same musical stimuli. A number of control tasks were carried out that allow evaluating the substance of the findings.

In line with the internal clock model (see Block et al., 2010), we assumed that individuals perceive duration to be shorter when attention is diverted, and hence fewer pulses are counted in the accumulator. Synchronizing with the beat of the music indeed shortened perceived durations compared to listening only. The dual-task condition, involving a math working-memory interference, led to the shortest duration estimates in absolute numbers; yet due to higher variance, estimates did not significantly differ from the other conditions. While this task diverted participants’ attention, their calculation success rate in the simple math task was not reflected in time experiences. Findings for perceived passage of time, on the other hand, support our hypothesis fully: the more demanding the task was, the quicker time passed subjectively. These results are further corroborated by the metronome control condition, which showed that tapping to the metronome compared to listening only to the metronome resulted in shorter perceived duration and quicker PoT. Hence the activity of tapping diverted attention to the motor task and increased cognitive load.

Results were not influenced by perceived arousal, as there were no main effects or interactions. The two high-arousal examples contained more events in terms of notes per seconds, but tempo was kept constant. Stimuli were reliably judged to be indeed higher in arousal, both by listeners in the pilot study and again by the participants of the main experiment. In contrast to our results, previous research found effects of subjectively felt arousal that increased duration estimates (Schwarz et al., 2013). A further study proposed that musical tempo is associated with higher perceived arousal and duration, while differences in musical orchestration only affected perceived arousal but did not lead to longer duration judgements (Droit-Volet et al., 2013). Since changes in musical tempo clearly also increase the number of events perceived, both tempo and/or arousal could result in the pacemaker emitting more pulses (Burle & Casini, 2001). Yet it is less known if this holds true for perceived arousal in stimuli or should involve a physiological component in the perceiver. Indeed, increasing the tempo of popular music songs increased skin conductance as a physiological measure of arousal (Kim, Strohbach, & Wedell, 2019), and manipulating individuals’ arousal may affect tempo selection tasks (Jakubowski et al., 2015). In the study by Schwarz et al. (2013), on the other hand, physiological decreases in heart-rate did not affect time judgements, in contrast to subjectively felt arousal as mentioned above. While it cannot be ruled out that increased cognitive load in our study may potentially also have affected physiological arousal, the working memory or tapping tasks were not considered to be overly demanding or arousing. Follow-up research could further disentangle the impact of participants’ subjectively felt arousal in relation to their physiological arousal (see, e.g., Schäfer & Sedlmeier, 2011), as well as the perceived arousal in the stimuli (as in Droit-Volet et al., 2013, and in the current study), with regards to their capacity for regulating the pulses in the pacemaker. In a previous audiovisual perception study, we found that individuals underestimated the duration of those stimuli more that were both rated lower in arousal and also yielded lower peripheral arousal responses in physiological markers such as heart rate, GSR and pupil size; in other words: low-arousal stimuli seemed to last shorter (Wöllner et al., 2018). Music is ideal for investigating these processes in a non-invasive and controlled way, and the impact of tempo and number of perceived events should be further scrutinized in relation to perceived arousal (in stimulus), subjectively felt arousal (of one’s body), and physiological arousal (as measured, e.g., in terms of heart rate or skin conductance).

Our previous finding about the influence of metrical levels (Hammerschmidt & Wöllner, 2020) was fully replicated for actual hip-hop music. Attending to a higher metrical level in the music, and thus tapping at a slower pace, may reduce the number of pulses emitted in the pacemaker and consequently shorten subjective time. These results suggest that engaging with music in an attentive way directly shapes time experiences. The effect was clearly related to perceiving the music in different ways, and does not simply reflect a different pulse due to the motor component in tapping. This result is again corroborated by the motor control condition, in which the speed at which participants tapped along with a metronome did not influence their duration estimates. As stated above, when comparing tapping to listening only to the metronome, tapping resulted in shorter duration estimates, which suggests that cognitive load was higher while tapping. Findings are different for perceived PoT, in that there were no significant effects of metrical level for the hip-hop music, while the higher tapping rate in the motor control condition led to faster PoT. It could well be that the flow of time during music listening was not perceived to be different, since the musical material itself remained the same. The metronome control task, in contrast, involved a higher number of beats in the high-tempo condition that affected concurrently perceived PoT. For perceived duration judgements, on the other hand, attending to different metrical levels in the music had a clear impact on how subjective time was filled, which was not simply due to the tempo differences as in the metronome tapping task. Taken together, investigating both prospective duration estimations and, in addition, perceived passage of time offers useful complementary insights in time dimensions that may differ depending on stimulus durations (Droit-Volet and Wearden, 2016; Droit-Volet et al., 2017).

Spontaneous motor tempo, averaged before and after the experiment, did not influence time judgements. The mean motor tempo was slower than the IOIs of 500–600 ms found in previous samples for preferred tempi (see McKinney & Moelants, 2006). It can be speculated that the pulse rate of an internal pacemaker is reflected in individuals’ spontaneous motor and preferred tempo (McAuley et al., 2006), but results so far remain inconclusive and warrant further research that should take into account factors such as living conditions, arousal or chronobiology. Contrary to previous research, familiarity with the music (Bailey & Areni, 2006; Lopez & Malhotra, 1991), preference (cf. Oakes, 2003) or musical experience (Grondin & Killeen, 2009) did not influence time judgements significantly. Similar to our previous study, musical experience did also not influence duration estimates using stimuli durations of similar length (Hammerschmidt & Wöllner, 2020).

A potential limitation is that the musical excerpts were relatively short, lasting from 20–31 s, depending on the number of bars (these varied systematically and were the same for low- and high-arousal examples). The different durations were correctly identified by participants. Experiences of time in music may, nevertheless, evolve differently over longer time spans, allowing for stronger expectations about metrical structure. Furthermore, when listening to music, individuals typically report flow and other experiences of temporal changes afterwards, which calls for more research using retrospective duration estimation paradigms. These experiences may differ for various musical genres. Tempo was deliberately held constant, and the slight digital manipulations to adjust overall tempo were not audible. Future research could vary tempo by controlling for arousal (cf. Droit-Volet et al., 2013) and vary cognitive load by asking participants to tap to more complex rhythms.

Taken together, this study used a paradigm that scrutinized the impact of cognitive load (higher load leads to shorter duration estimates), musical meter (higher metrical level leads to shorter durations estimates), and arousal in the music (no influence), while keeping tempo constant. These findings were stable irrespective of individual differences between participants. They add further facets to psychological theories of time experiences and warrant more research on the role of perceived and felt arousal in relation to tempo. Finally, if individuals dancing to hip-hop (and other music) should wish the moment to linger for longer, then they should attend to lower metrical levels and synchronize at a higher pace. In this regard, the slow hip-hop tempi also afford dancing at twice the speed.

## References

[CR1] Bailey N, Areni CS (2006). When a few minutes sound like a lifetime: Does atmospheric music expand or contract perceived time?. Journal of Retailing.

[CR2] Block RA, Hancock PA, Zakay D (2010). How cognitive load affects duration judgements: A meta-analytic review. Acta Psychologica.

[CR3] Block RA, Zakay D (1997). Prospective and retrospective duration judgements: A meta- analytic review. Psychonomic Bulletin & Review.

[CR4] Boltz, M. G. (1989). Time judgements of musical endings: Effects of expectancies on the “filled interval effect”. *Perception & Psychophysics, 46,* 409–418. 10.3758/BF03210855 10.3758/bf03210855 2813025

[CR5] Brown SW, Boltz MG (2002). Attentional processes in time perception: Effects of mental workload and event structure. Journal of Experimental Psychology: Human Perception and Performance.

[CR6] Bueno, J. L. O., Firmino, É. A. & Engelman, A. (2002). Influence of generalized complexity of a musical event on subjective time estimation. *Perceptual and Motor Skills*, *94*, 541–547. 10.2466/pms.2002.94.2.541 10.2466/pms.2002.94.2.541 12027350

[CR7] Burger B, London J, Thompson MR, Toiviainen P (2018). Synchronization to metrical levels in music depends on low-frequency spectral components and tempo. Psychological Research.

[CR8] Burle, B. S., & Casini, L. (2001). Dissociation between activation and attention effects in time estimation: Implications for internal clock models. *Journal of Experimental Psychology: Human Perception and Performance, 27*(1)*,* 195–205. 10.1037/0096-1523.27.1.195 10.1037//0096-1523.27.1.195 11248933

[CR9] Drake C, Ben El Heni J (2003). Synchronizing with music: Intercultural differences. Annals of the New York Academy of Science.

[CR10] Drake, C., Jones, M. R., & Baruch, C. (2000). The development of rhythmic attending in auditory sequences: Attunement, referent period, focal attending. *Cognition*, *77*(3), 251–288. 10.1016/S0010-0277(00)00106-2 10.1016/s0010-0277(00)00106-2 11018511

[CR11] Drake, C., Penel, A., & Bigand, E. (2000). Tapping in time with mechanically and expressively performed music. *Music Perception*, *18*(1), 1–23. 10.2307/40285899

[CR12] Droit-Volet, S., Ramos, D., Bueno, J. L. O., & Bigand, E. (2013). Music, emotion, and time perception: The influence of subjective emotional valence and arousal? *Frontiers in Psychology,* 4(417)*.*10.3389/fpsyg.2013.00417 10.3389/fpsyg.2013.00417 PMC3713348 23882233

[CR13] Droit-Volet, S., Trahanias, P., & Maniadakis, M. (2017). Passage of time judgements in everyday life are not related to duration judgements except for long durations of several minutes. *Acta Psychologica, 73,* 116–121. 10.1016/j.actpsy.2016.12.010 10.1016/j.actpsy.2016.12.010 28040645

[CR14] Droit-Volet, S., & Wearden, J. (2016). Passage of time judgements are not duration judgements: Evidence from a study using experience sampling methodology. *Frontiers in Psychology, 7*(176). 10.3389/fpsyg.2016.00176 10.3389/fpsyg.2016.00176 PMC4759643 26925006

[CR15] Faul, F., Erdfelder, E., Lang, A. , & Buchner, A. (2007). G*Power 3: A flexible statistical power analysis program for the social, behavioral, and biomedical sciences. *Behavior Research Methods, 39,* 175–191. 10.3758/BF03193146 10.3758/bf03193146 17695343

[CR16] Gibbon J (1991). Origins of scalar timing. Learning and Motivation.

[CR17] Grondin S, Killeen PR (2009). Tracking time with song and count: Different Weber functions for musicians and nonmusicians. Attention, Perception, & Psychophysics.

[CR18] Hammerschmidt D, Wöllner C (2020). Sensorimotor synchronization with higher metrical levels in music shortens perceived time. Music Perception.

[CR19] Jakubowski K, Halpern AR, Grierson M, Stuart L (2015). The effect of exercise-induced arousal on chosen tempi for familiar melodies. Psychonomic Bulletin and Review.

[CR20] Kim J, Strohbach CA, Wedell DH (2019). Effects of manipulating the tempo of popular songs on behavioral and physiological responses. Psychology of Music.

[CR21] Lartillot O, Toiviainen P, Eerola T, Preisach C, Burkhardt H, Schmidt-Thieme L, Decker R (2008). A Matlab toolbox for Music Information Retrieval. *Data analysis, machine learning and applications*.

[CR22] Leight, E. (2017). Producers, songwriters on how pop songs got so slow.* Rolling Stone,* August 15, 2017. https://www.rollingstone.com/music/music-features/how-did-pop-music-get-so-slow-197794

[CR23] London J (2002). Cognitive constraints on metric systems: Some observations and hypotheses. Music Perception.

[CR24] Lopez L, Malhotra R (1991). Estimation of time intervals with most preferred and least preferred music. Psychological Studies.

[CR25] Mathôt S, Schreij D, Theuwes J (2012). OpenSesame: An open-source, graphical experiment builder for the social sciences. Behavior Research Methods.

[CR26] McAuley JD, Jones MR, Holub S, Johnston HM, Miller NS (2006). The time of our lives: Life span development of timing and event tracking. Journal of Experimental Psychology: General.

[CR27] McKinney, M. F., & Moelants, D. (2006). Ambiguity in tempo perception: What draws listeners to different metrical levels? *Music Perception, 24*(2), 155–165. 10.1525/mp.2006.24.2.155

[CR28] Noulhiane M, Mella N, Samson S, Ragot R, Pouthas V (2007). How emotional auditory stimuli modulate time perception. Emotion.

[CR29] Oakes S (2003). Musical tempo and waiting perceptions. Psychology & Marketing.

[CR30] Polti I, Martin B, van Wassenhove V (2018). The effect of attention and working memory on the estimation of elapsed time. Scientific Reports.

[CR31] Repp BH (2010). Sensorimotor synchronization and perception of timing: Effects of music training and task experience. Human Movement Science.

[CR32] Schäfer T, Sedlmeier P (2011). Does the body move the soul? The impact of arousal on music preference. Music Perception.

[CR33] Schwarz, M. A., Winkler, I., & Sedlmeier, P. (2013). The heart beat does not make us tick: The impacts of heart rate and arousal on time perception. *Attention, Perception, & Psychophysics, 75,* 182–193. 10.3758/s13414-012-0387-8 10.3758/s13414-012-0387-8 23143915

[CR34] Snyder J, Krumhansl CL (2001). Tapping to ragtime: Cues to pulse finding. Music Perception.

[CR35] Teki S, Griffiths TD (2014). Working memory for time intervals in auditory rhythmic sequences. Frontiers in Psychology.

[CR36] van Wassenhove V (2016). Temporal cognition and neural oscillations. Current Opinion in Behavioral Sciences.

[CR37] Wearden JH (2008). Slowing down an internal clock: Implications for accounts of performance on four timing tasks. Quarterly Journal of Experimental Psychology.

[CR38] Wittmann, M., Simmons, A.N., Aron, J., & Paulus, M.P. (2010). Accumulation of neural activity in the posterior insula encodes the passage of time. *Neuropsychologia, 48,* 3110–3120. 10.1016/j.neuropsychologia.2010.06.023 10.1016/j.neuropsychologia.2010.06.023 PMC2933788 20600186

[CR39] Wöllner C, Hammerschmidt D, Albrecht H (2018). Slow motion in films and video clips: Music influences perceived duration and emotion, autonomic physiological activation and pupillary responses. PLoS ONE.

